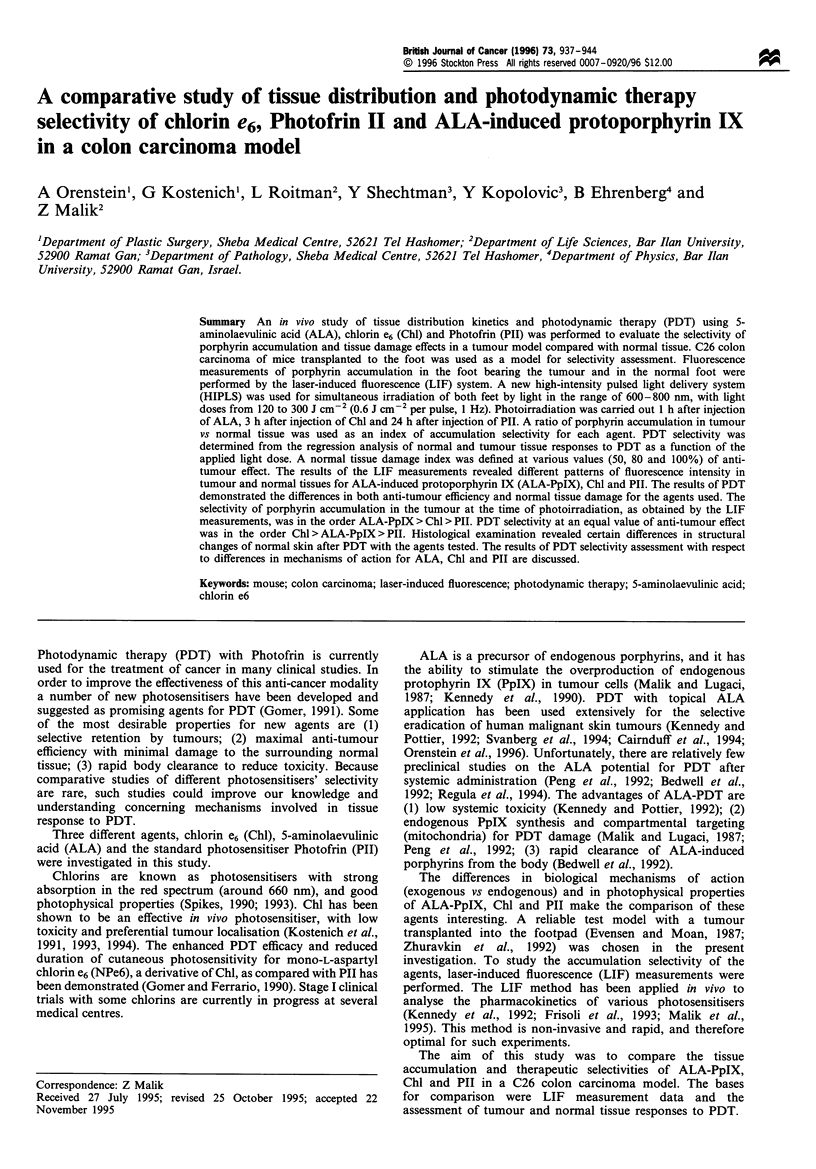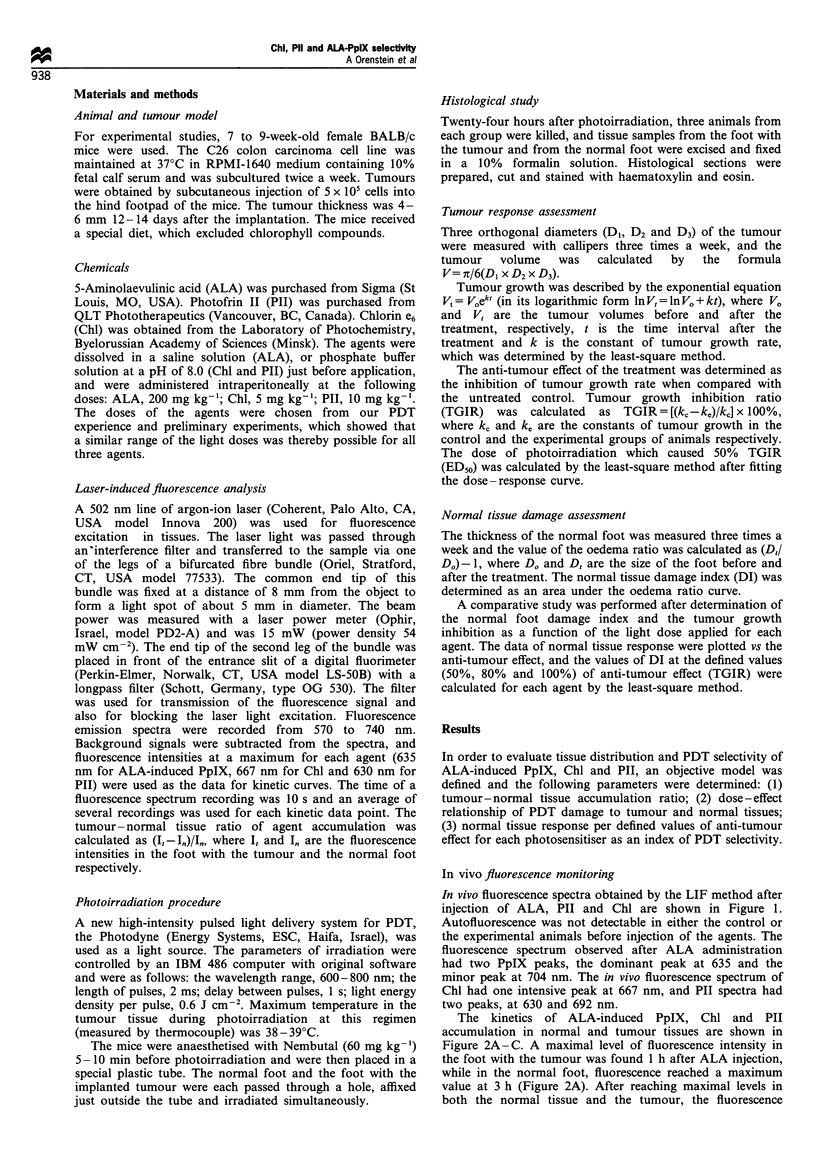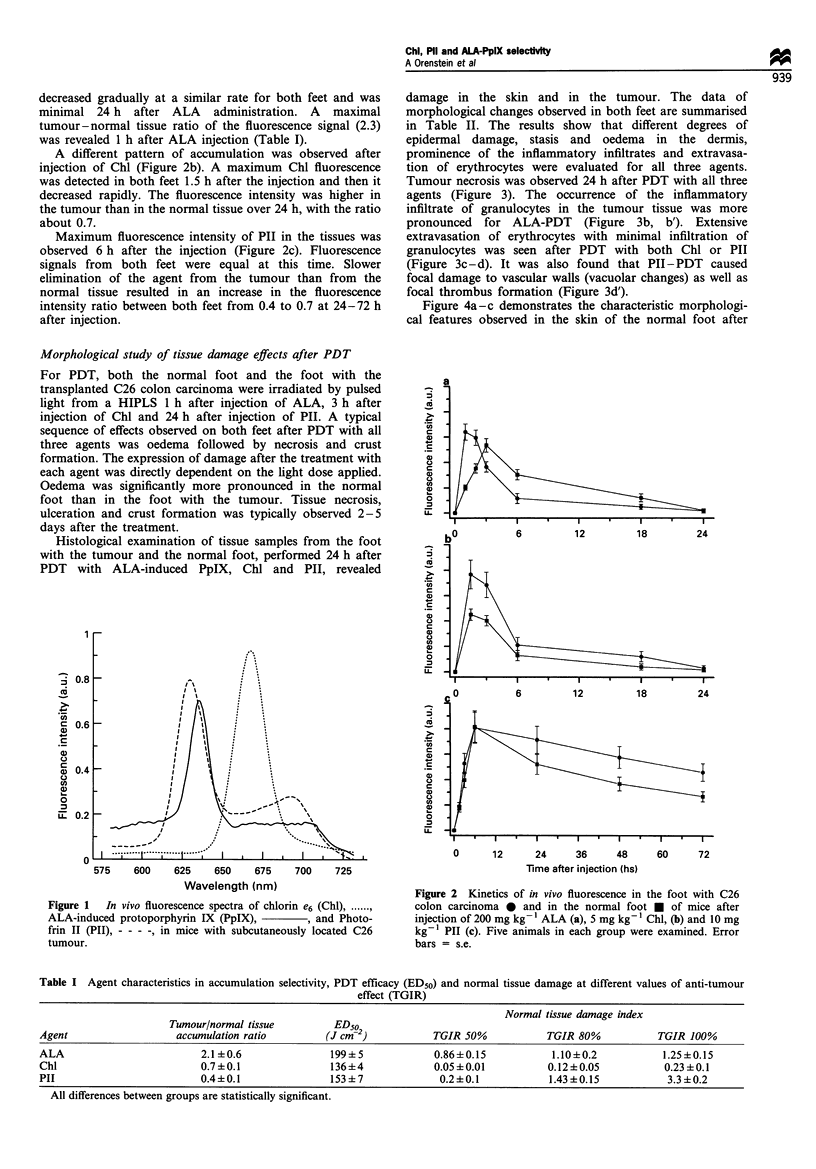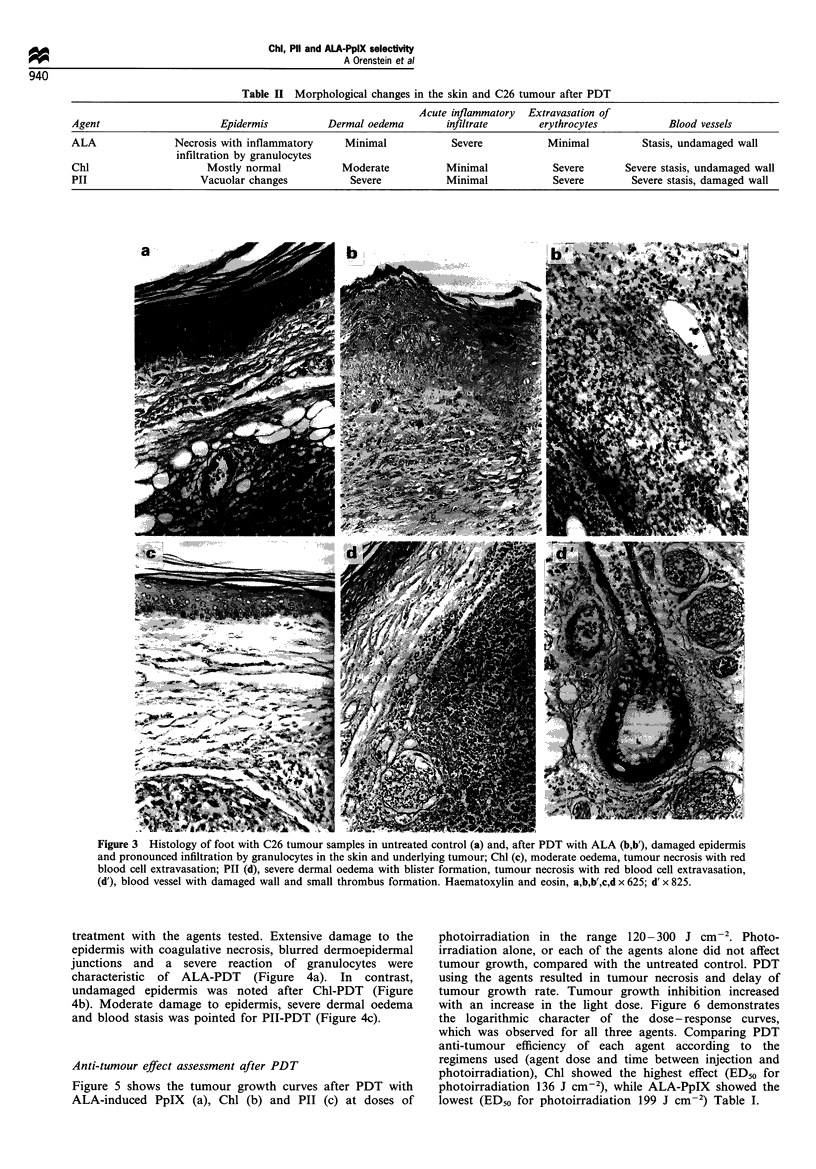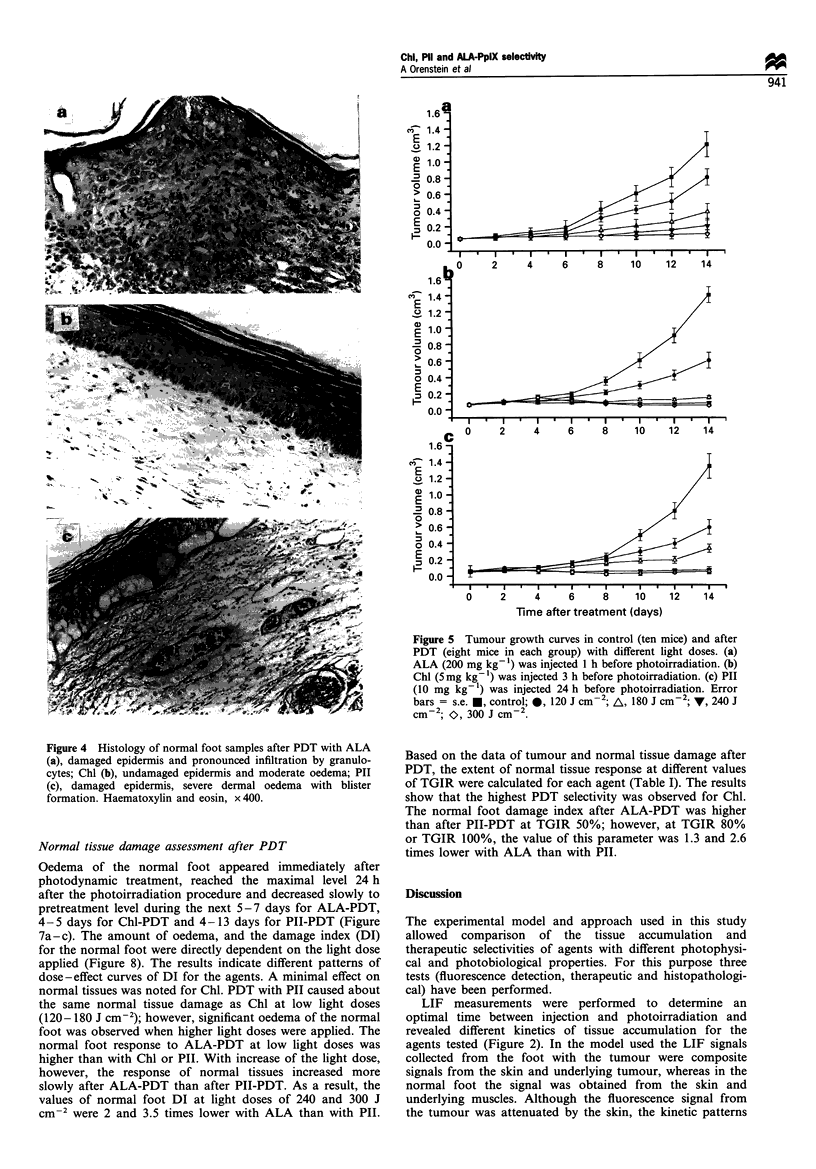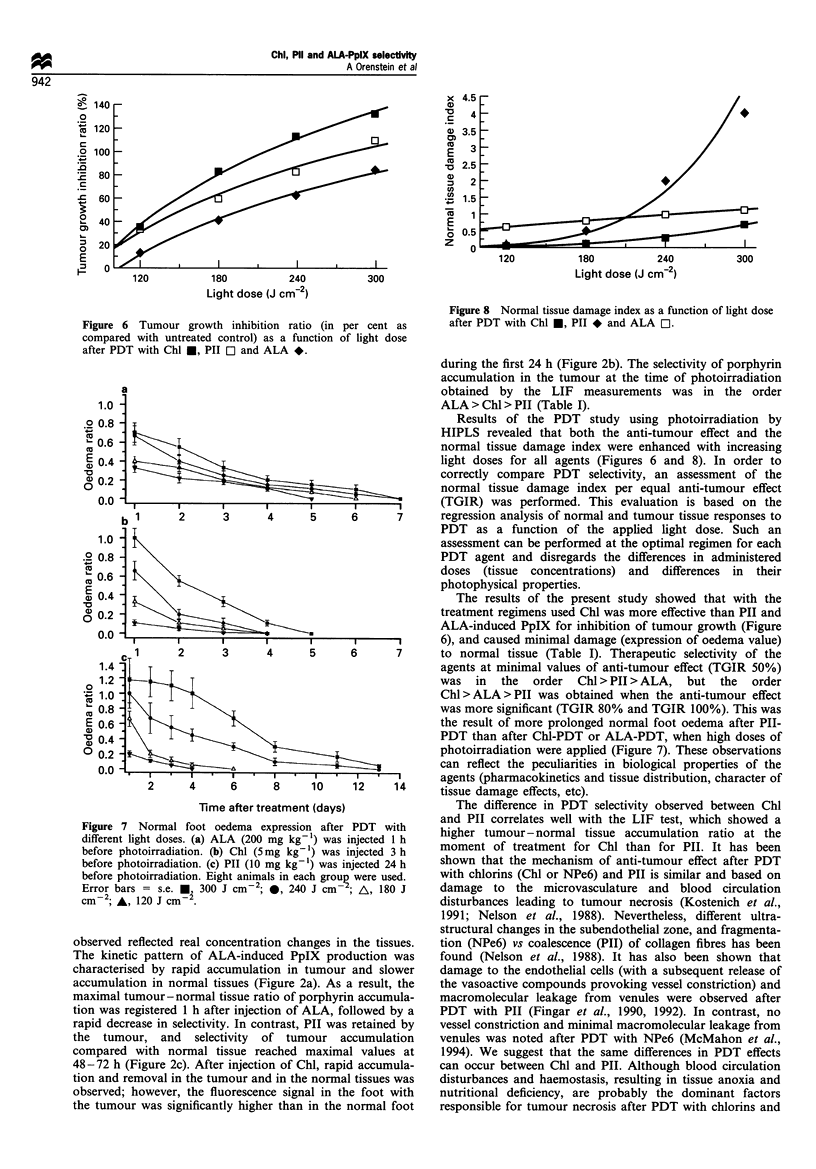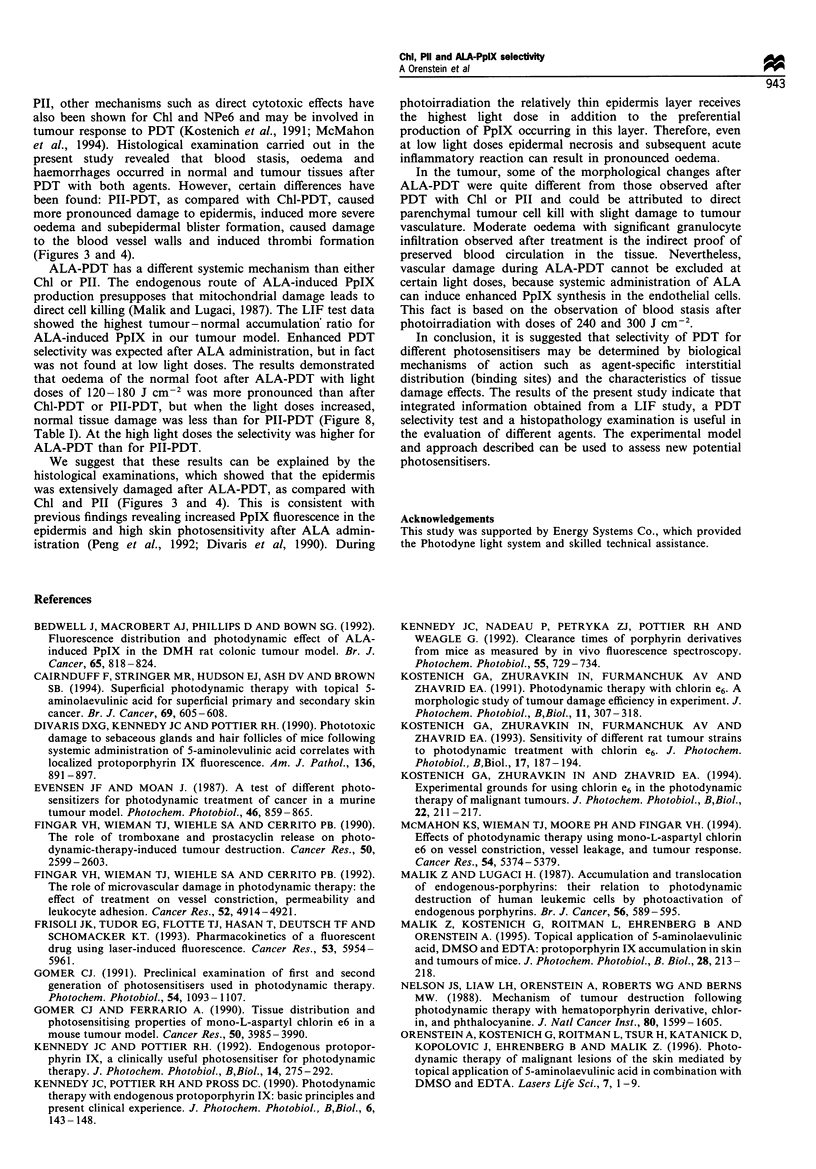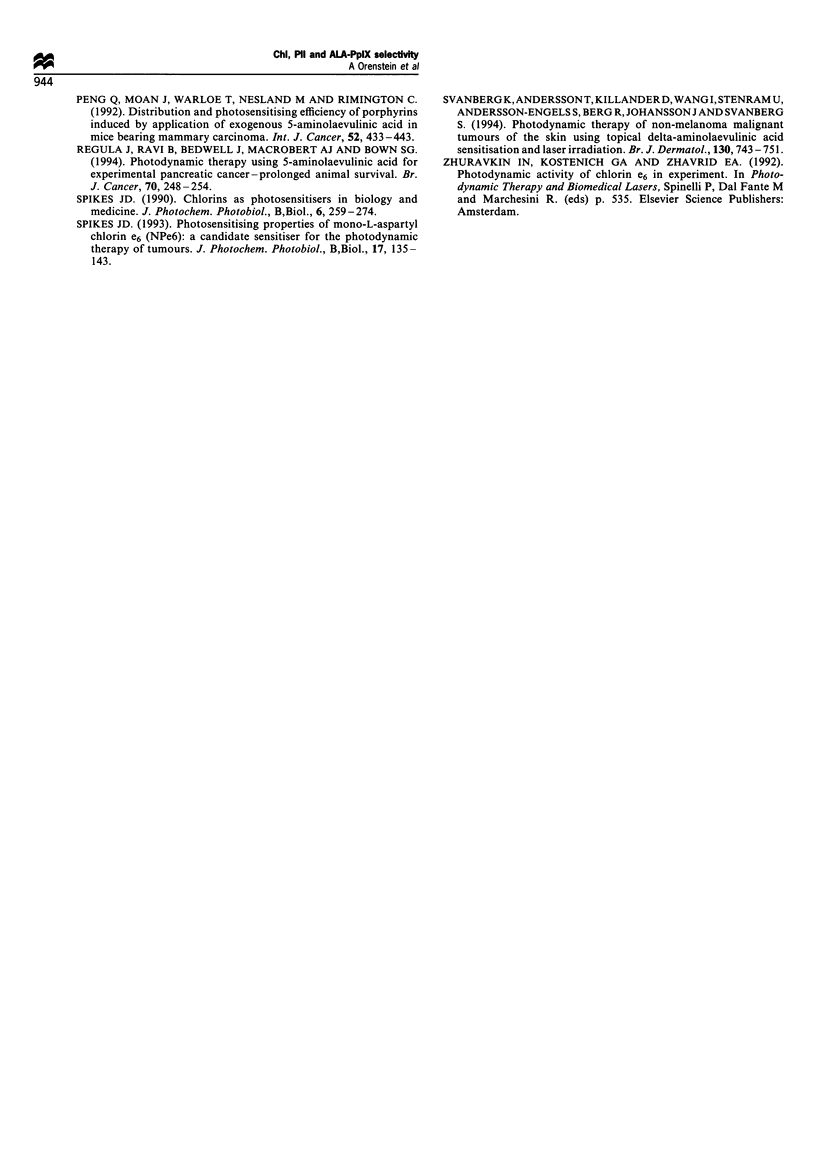# A comparative study of tissue distribution and photodynamic therapy selectivity of chlorin e6, Photofrin II and ALA-induced protoporphyrin IX in a colon carcinoma model.

**DOI:** 10.1038/bjc.1996.185

**Published:** 1996-04

**Authors:** A. Orenstein, G. Kostenich, L. Roitman, Y. Shechtman, Y. Kopolovic, B. Ehrenberg, Z. Malik

**Affiliations:** Department of Plastic Surgery, Sheba Medical Centre, Tel Hashomer, Israel.

## Abstract

**Images:**


British Journal of Cancer (1996) 73, 937-944
© 1996 Stockton Press All rights reserved 0007-0920/96 $12.00

### A comparative study of tissue distribution and photodynamic therapy selectivity of chlorin $e_6$, Photofrin II and ALA-induced protoporphyrin IX in a colon carcinoma model

A Orenstein[1], G Kostenich[1], L Roitman[2], Y Shechtman[3], Y Kopolovic[3], B Ehrenberg[4] and Z Malik[2]

[1]*Department of Plastic Surgery, Sheba Medical Centre, 52621 Tel Hashomer;* [2]*Department of Life Sciences, Bar Ilan University, 52900 Ramat Gan;* [3]*Department of Pathology, Sheba Medical Centre, 52621 Tel Hashomer,* [4]*Department of Physics, Bar Ilan University, 52900 Ramat Gan, Israel.*

**Summary** An *in vivo* study of tissue distribution kinetics and photodynamic therapy (PDT) using 5-aminolaevulinic acid (ALA), chlorin $e_6$ (Chl) and Photofrin (PII) was performed to evaluate the selectivity of porphyrin accumulation and tissue damage effects in a tumour model compared with normal tissue. C26 colon carcinoma of mice transplanted to the foot was used as a model for selectivity assessment. Fluorescence measurements of porphyrin accumulation in the foot bearing the tumour and in the normal foot were performed by the laser-induced fluorescence (LIF) system. A new high-intensity pulsed light delivery system (HIPLS) was used for simultaneous irradiation of both feet by light in the range of 600-800 nm, with light doses from 120 to 300 J $cm^{-2}$ (0.6 J $cm^{-2}$ per pulse, 1 Hz). Photoirradiation was carried out 1 h after injection of ALA, 3 h after injection of Chl and 24 h after injection of PII. A ratio of porphyrin accumulation in tumour *vs* normal tissue was used as an index of accumulation selectivity for each agent. PDT selectivity was determined from the regression analysis of normal and tumour tissue responses to PDT as a function of the applied light dose. A normal tissue damage index was defined at various values (50, 80 and 100%) of anti-tumour effect. The results of the LIF measurements revealed different patterns of fluorescence intensity in tumour and normal tissues for ALA-induced protoporphyrin IX (ALA-PpIX), Chl and PII. The results of PDT demonstrated the differences in both anti-tumour efficiency and normal tissue damage for the agents used. The selectivity of porphyrin accumulation in the tumour at the time of photoirradiation, as obtained by the LIF measurements, was in the order ALA-PpIX > Chl > PII. PDT selectivity at an equal value of anti-tumour effect was in the order Chl > ALA-PpIX > PII. Histological examination revealed certain differences in structural changes of normal skin after PDT with the agents tested. The results of PDT selectivity assessment with respect to differences in mechanisms of action for ALA, Chl and PII are discussed.

**Keywords:** mouse; colon carcinoma; laser-induced fluorescence; photodynamic therapy; 5-aminolaevulinic acid; chlorin e6

Photodynamic therapy (PDT) with Photofrin is currently used for the treatment of cancer in many clinical studies. In order to improve the effectiveness of this anti-cancer modality a number of new photosensitisers have been developed and suggested as promising agents for PDT (Gomer, 1991). Some of the most desirable properties for new agents are (1) selective retention by tumours; (2) maximal anti-tumour efficiency with minimal damage to the surrounding normal tissue; (3) rapid body clearance to reduce toxicity. Because comparative studies of different photosensitisers' selectivity are rare, such studies could improve our knowledge and understanding concerning mechanisms involved in tissue response to PDT.

Three different agents, chlorin $e_6$ (Chl), 5-aminolaevulinic acid (ALA) and the standard photosensitiser Photofrin (PII) were investigated in this study.

Chlorins are known as photosensitisers with strong absorption in the red spectrum (around 660 nm), and good photophysical properties (Spikes, 1990; 1993). Chl has been shown to be an effective *in vivo* photosensitiser, with low toxicity and preferential tumour localisation (Kostenich *et al.*, 1991, 1993, 1994). The enhanced PDT efficacy and reduced duration of cutaneous photosensitivity for mono-L-aspartyl chlorin $e_6$ (NPe6), a derivative of Chl, as compared with PII has been demonstrated (Gomer and Ferrario, 1990). Stage I clinical trials with some chlorins are currently in progress at several medical centres.

Correspondence: Z Malik
Received 27 July 1995; revised 25 October 1995; accepted 22 November 1995

ALA is a precursor of endogenous porphyrins, and it has the ability to stimulate the overproduction of endogenous protophyrin IX (PpIX) in tumour cells (Malik and Lugaci, 1987; Kennedy *et al.*, 1990). PDT with topical ALA application has been used extensively for the selective eradication of human malignant skin tumours (Kennedy and Pottier, 1992; Svanberg *et al.*, 1994; Cairnduff *et al.*, 1994; Orenstein *et al.*, 1996). Unfortunately, there are relatively few preclinical studies on the ALA potential for PDT after systemic administration (Peng *et al.*, 1992; Bedwell *et al.*, 1992; Regula *et al.*, 1994). The advantages of ALA-PDT are (1) low systemic toxicity (Kennedy and Pottier, 1992); (2) endogenous PpIX synthesis and compartmental targeting (mitochondria) for PDT damage (Malik and Lugaci, 1987; Peng *et al.*, 1992; (3) rapid clearance of ALA-induced porphyrins from the body (Bedwell *et al.*, 1992).

The differences in biological mechanisms of action (exogenous *vs* endogenous) and in photophysical properties of ALA-PpIX, Chl and PII make the comparison of these agents interesting. A reliable test model with a tumour transplanted into the footpad (Evensen and Moan, 1987; Zhuravkin *et al.*, 1992) was chosen in the present investigation. To study the accumulation selectivity of the agents, laser-induced fluorescence (LIF) measurements were performed. The LIF method has been applied *in vivo* to analyse the pharmacokinetics of various photosensitisers (Kennedy *et al.*, 1992; Frisoli *et al.*, 1993; Malik *et al.*, 1995). This method is non-invasive and rapid, and therefore optimal for such experiments.

The aim of this study was to compare the tissue accumulation and therapeutic selectivities of ALA-PpIX, Chl and PII in a C26 colon carcinoma model. The bases for comparison were LIF measurement data and the assessment of tumour and normal tissue responses to PDT.

### Materials and methods

*Animal and tumour model*

For experimental studies, 7 to 9-week-old female BALB/c mice were used. The C26 colon carcinoma cell line was maintained at 37°C in RPMI-1640 medium containing 10% fetal calf serum and was subcultured twice a week. Tumours were obtained by subcutaneous injection of $5 \times 10^5$ cells into the hind footpad of the mice. The tumour thickness was 4–6 mm 12–14 days after the implantation. The mice received a special diet, which excluded chlorophyll compounds.

*Chemicals*

5-Aminolaevulinic acid (ALA) was purchased from Sigma (St Louis, MO, USA). Photofrin II (PII) was purchased from QLT Phototherapeutics (Vancouver, BC, Canada). Chlorin $e_6$ (Chl) was obtained from the Laboratory of Photochemistry, Byelorussian Academy of Sciences (Minsk). The agents were dissolved in a saline solution (ALA), or phosphate buffer solution at a pH of 8.0 (Chl and PII) just before application, and were administered intraperitoneally at the following doses: ALA, 200 mg $kg^{-1}$; Chl, 5 mg $kg^{-1}$; PII, 10 mg $kg^{-1}$. The doses of the agents were chosen from our PDT experience and preliminary experiments, which showed that a similar range of the light doses was thereby possible for all three agents.

*Laser-induced fluorescence analysis*

A 502 nm line of argon-ion laser (Coherent, Palo Alto, CA, USA model Innova 200) was used for fluorescence excitation in tissues. The laser light was passed through an interference filter and transferred to the sample via one of the legs of a bifurcated fibre bundle (Oriel, Stratford, CT, USA model 77533). The common end tip of this bundle was fixed at a distance of 8 mm from the object to form a light spot of about 5 mm in diameter. The beam power was measured with a laser power meter (Ophir, Israel, model PD2-A) and was 15 mW (power density 54 mW $cm^{-2}$). The end tip of the second leg of the bundle was placed in front of the entrance slit of a digital fluorimeter (Perkin-Elmer, Norwalk, CT, USA model LS-50B) with a longpass filter (Schott, Germany, type OG 530). The filter was used for transmission of the fluorescence signal and also for blocking the laser light excitation. Fluorescence emission spectra were recorded from 570 to 740 nm. Background signals were subtracted from the spectra, and fluorescence intensities at a maximum for each agent (635 nm for ALA-induced PpIX, 667 nm for Chl and 630 nm for PII) were used as the data for kinetic curves. The time of a fluorescence spectrum recording was 10 s and an average of several recordings was used for each kinetic data point. The tumour–normal tissue ratio of agent accumulation was calculated as $(I_t - I_n)/I_n$, where $I_t$ and $I_n$ are the fluorescence intensities in the foot with the tumour and the normal foot respectively.

*Photoirradiation procedure*

A new high-intensity pulsed light delivery system for PDT, the Photodyne (Energy Systems, ESC, Haifa, Israel), was used as a light source. The parameters of irradiation were controlled by an IBM 486 computer with original software and were as follows: the wavelength range, 600–800 nm; the length of pulses, 2 ms; delay between pulses, 1 s; light energy density per pulse, 0.6 J $cm^{-2}$. Maximum temperature in the tumour tissue during photoirradiation at this regimen (measured by thermocouple) was 38–39°C.

The mice were anaesthetised with Nembutal (60 mg $kg^{-1}$) 5–10 min before photoirradiation and were then placed in a special plastic tube. The normal foot and the foot with the implanted tumour were each passed through a hole, affixed just outside the tube and irradiated simultaneously.

*Histological study*

Twenty-four hours after photoirradiation, three animals from each group were killed, and tissue samples from the foot with the tumour and from the normal foot were excised and fixed in a 10% formalin solution. Histological sections were prepared, cut and stained with haematoxylin and eosin.

*Tumour response assessment*

Three orthogonal diameters ($D_1$, $D_2$ and $D_3$) of the tumour were measured with callipers three times a week, and the tumour volume was calculated by the formula $V = \pi/6(D_1 \times D_2 \times D_3)$.

Tumour growth was described by the exponential equation $V_t = V_o e^{kt}$ (in its logarithmic form $\ln V_t = \ln V_o + kt$), where $V_o$ and $V_t$ are the tumour volumes before and after the treatment, respectively, $t$ is the time interval after the treatment and $k$ is the constant of tumour growth rate, which was determined by the least-square method.

The anti-tumour effect of the treatment was determined as the inhibition of tumour growth rate when compared with the untreated control. Tumour growth inhibition ratio (TGIR) was calculated as $TGIR = [(k_c - k_e)/k_c] \times 100\%$, where $k_c$ and $k_e$ are the constants of tumour growth in the control and the experimental groups of animals respectively. The dose of photoirradiation which caused 50% TGIR ($ED_{50}$) was calculated by the least-square method after fitting the dose–response curve.

*Normal tissue damage assessment*

The thickness of the normal foot was measured three times a week and the value of the oedema ratio was calculated as $(D_t/D_o) - 1$, where $D_o$ and $D_t$ are the size of the foot before and after the treatment. The normal tissue damage index (DI) was determined as an area under the oedema ratio curve.

A comparative study was performed after determination of the normal foot damage index and the tumour growth inhibition as a function of the light dose applied for each agent. The data of normal tissue response were plotted *vs* the anti-tumour effect, and the values of DI at the defined values (50%, 80% and 100%) of anti-tumour effect (TGIR) were calculated for each agent by the least-square method.

### Results

In order to evaluate tissue distribution and PDT selectivity of ALA-induced PpIX, Chl and PII, an objective model was defined and the following parameters were determined: (1) tumour–normal tissue accumulation ratio; (2) dose–effect relationship of PDT damage to tumour and normal tissues; (3) normal tissue response per defined values of anti-tumour effect for each photosensitiser as an index of PDT selectivity.

In vivo *fluorescence monitoring*

*In vivo* fluorescence spectra obtained by the LIF method after injection of ALA, PII and Chl are shown in Figure 1. Autofluorescence was not detectable in either the control or the experimental animals before injection of the agents. The fluorescence spectrum observed after ALA administration had two PpIX peaks, the dominant peak at 635 and the minor peak at 704 nm. The *in vivo* fluorescence spectrum of Chl had one intensive peak at 667 nm, and PII spectra had two peaks, at 630 and 692 nm.

The kinetics of ALA-induced PpIX, Chl and PII accumulation in normal and tumour tissues are shown in Figure 2A–C. A maximal level of fluorescence intensity in the foot with the tumour was found 1 h after ALA injection, while in the normal foot, fluorescence reached a maximum value at 3 h (Figure 2A). After reaching maximal levels in both the normal tissue and the tumour, the fluorescence decreased gradually at a similar rate for both feet and was minimal 24 h after ALA administration. A maximal tumour–normal tissue ratio of the fluorescence signal (2.3) was revealed 1 h after ALA injection (Table I).

A different pattern of accumulation was observed after injection of Chl (Figure 2b). A maximum Chl fluorescence was detected in both feet 1.5 h after the injection and then it decreased rapidly. The fluorescence intensity was higher in the tumour than in the normal tissue over 24 h, with the ratio about 0.7.

Maximum fluorescence intensity of PII in the tissues was observed 6 h after the injection (Figure 2c). Fluorescence signals from both feet were equal at this time. Slower elimination of the agent from the tumour than from the normal tissue resulted in an increase in the fluorescence intensity ratio between both feet from 0.4 to 0.7 at 24–72 h after injection.

### *Morphological study of tissue damage effects after PDT*

For PDT, both the normal foot and the foot with the transplanted C26 colon carcinoma were irradiated by pulsed light from a HIPLS 1 h after injection of ALA, 3 h after injection of Chl and 24 h after injection of PII. A typical sequence of effects observed on both feet after PDT with all three agents was oedema followed by necrosis and crust formation. The expression of damage after the treatment with each agent was directly dependent on the light dose applied. Oedema was significantly more pronounced in the normal foot than in the foot with the tumour. Tissue necrosis, ulceration and crust formation was typically observed 2–5 days after the treatment.

Histological examination of tissue samples from the foot with the tumour and the normal foot, performed 24 h after PDT with ALA-induced PpIX, Chl and PII, revealed damage in the skin and in the tumour. The data of morphological changes observed in both feet are summarised in Table II. The results show that different degrees of epidermal damage, stasis and oedema in the dermis, prominence of the inflammatory infiltrates and extravasation of erythrocytes were evaluated for all three agents. Tumour necrosis was observed 24 h after PDT with all three agents (Figure 3). The occurrence of the inflammatory infiltrate of granulocytes in the tumour tissue was more pronounced for ALA-PDT (Figure 3b, b′). Extensive extravasation of erythrocytes with minimal infiltration of granulocytes was seen after PDT with both Chl or PII (Figure 3c–d). It was also found that PII–PDT caused focal damage to vascular walls (vacuolar changes) as well as focal thrombus formation (Figure 3d′).

Figure 4a–c demonstrates the characteristic morphological features observed in the skin of the normal foot after

**Figure 1** *In vivo* fluorescence spectra of chlorin $e_6$ (Chl), ......, ALA-induced protoporphyrin IX (PpIX), ————, and Photofrin II (PII), - - - -, in mice with subcutaneously located C26 tumour.

**Figure 2** Kinetics of *in vivo* fluorescence in the foot with C26 colon carcinoma ● and in the normal foot ■ of mice after injection of 200 mg $kg^{-1}$ ALA (**a**), 5 mg $kg^{-1}$ Chl, (**b**) and 10 mg $kg^{-1}$ PII (**c**). Five animals in each group were examined. Error bars = s.e.

**Table I** Agent characteristics in accumulation selectivity, PDT efficacy ($ED_{50}$) and normal tissue damage at different values of anti-tumour effect (TGIR)

| *Agent* | *Tumour/normal tissue accumulation ratio* | *$ED_{50}$ ($J\ cm^{-2}$)* | *Normal tissue damage index* TGIR 50% | *TGIR 80%* | *TGIR 100%* |
|---|---|---|---|---|---|
| ALA | 2.1±0.6 | 199±5 | 0.86±0.15 | 1.10±0.2 | 1.25±0.15 |
| Chl | 0.7±0.1 | 136±4 | 0.05±0.01 | 0.12±0.05 | 0.23±0.1 |
| PII | 0.4±0.1 | 153±7 | 0.2±0.1 | 1.43±0.15 | 3.3±0.2 |

All differences between groups are statistically significant.

**Table II** Morphological changes in the skin and C26 tumour after PDT

| *Agent* | *Epidermis* | *Dermal oedema* | *Acute inflammatory infiltrate* | *Extravasation of erythrocytes* | *Blood vessels* |
|---|---|---|---|---|---|
| ALA | Necrosis with inflammatory infiltration by granulocytes | Minimal | Severe | Minimal | Stasis, undamaged wall |
| Chl | Mostly normal | Moderate | Minimal | Severe | Severe stasis, undamaged wall |
| PII | Vacuolar changes | Severe | Minimal | Severe | Severe stasis, damaged wall |

**Figure 3** Histology of foot with C26 tumour samples in untreated control (**a**) and, after PDT with ALA (**b**,**b'**), damaged epidermis and pronounced infiltration by granulocytes in the skin and underlying tumour; Chl (**c**), moderate oedema, tumour necrosis with red blood cell extravasation; PII (**d**), severe dermal oedema with blister formation, tumour necrosis with red blood cell extravasation, (**d'**), blood vessel with damaged wall and small thrombus formation. Haematoxylin and eosin, **a**,**b**,**b'**,**c**,**d** × 625; **d'** × 825.

treatment with the agents tested. Extensive damage to the epidermis with coagulative necrosis, blurred dermoepidermal junctions and a severe reaction of granulocytes were characteristic of ALA-PDT (Figure 4a). In contrast, undamaged epidermis was noted after Chl-PDT (Figure 4b). Moderate damage to epidermis, severe dermal oedema and blood stasis was pointed for PII-PDT (Figure 4c).

### *Anti-tumour effect assessment after PDT*

Figure 5 shows the tumour growth curves after PDT with ALA-induced PpIX (a), Chl (b) and PII (c) at doses of photoirradiation in the range 120–300 J $cm^{-2}$. Photoirradiation alone, or each of the agents alone did not affect tumour growth, compared with the untreated control. PDT using the agents resulted in tumour necrosis and delay of tumour growth rate. Tumour growth inhibition increased with an increase in the light dose. Figure 6 demonstrates the logarithmic character of the dose–response curves, which was observed for all three agents. Comparing PDT anti-tumour efficiency of each agent according to the regimens used (agent dose and time between injection and photoirradiation), Chl showed the highest effect ($ED_{50}$ for photoirradiation 136 J $cm^{-2}$), while ALA-PpIX showed the lowest ($ED_{50}$ for photoirradiation 199 J $cm^{-2}$) Table I.

Figure 4 Histology of normal foot samples after PDT with ALA (a), damaged epidermis and pronounced infiltration by granulocytes; Chl (b), undamaged epidermis and moderate oedema; PII (c), damaged epidermis, severe dermal oedema with blister formation. Haematoxylin and eosin, ×400.

*Normal tissue damage assessment after PDT*

Oedema of the normal foot appeared immediately after photodynamic treatment, reached the maximal level 24 h after the photoirradiation procedure and decreased slowly to pretreatment level during the next 5–7 days for ALA-PDT, 4–5 days for Chl-PDT and 4–13 days for PII-PDT (Figure 7a–c). The amount of oedema, and the damage index (DI) for the normal foot were directly dependent on the light dose applied (Figure 8). The results indicate different patterns of dose–effect curves of DI for the agents. A minimal effect on normal tissues was noted for Chl. PDT with PII caused about the same normal tissue damage as Chl at low light doses (120–180 J $cm^{-2}$); however, significant oedema of the normal foot was observed when higher light doses were applied. The normal foot response to ALA-PDT at low light doses was higher than with Chl or PII. With increase of the light dose, however, the response of normal tissues increased more slowly after ALA-PDT than after PII-PDT. As a result, the values of normal foot DI at light doses of 240 and 300 J $cm^{-2}$ were 2 and 3.5 times lower with ALA than with PII.

Figure 5 Tumour growth curves in control (ten mice) and after PDT (eight mice in each group) with different light doses. (a) ALA (200 mg $kg^{-1}$) was injected 1 h before photoirradiation. (b) Chl (5 mg $kg^{-1}$) was injected 3 h before photoirradiation. (c) PII (10 mg $kg^{-1}$) was injected 24 h before photoirradiation. Error bars = s.e. ■, control; ●, 120 J $cm^{-2}$; △, 180 J $cm^{-2}$; ▼, 240 J $cm^{-2}$; ◇, 300 J $cm^{-2}$.

Based on the data of tumour and normal tissue damage after PDT, the extent of normal tissue response at different values of TGIR were calculated for each agent (Table I). The results show that the highest PDT selectivity was observed for Chl. The normal foot damage index after ALA-PDT was higher than after PII-PDT at TGIR 50%; however, at TGIR 80% or TGIR 100%, the value of this parameter was 1.3 and 2.6 times lower with ALA than with PII.

### Discussion

The experimental model and approach used in this study allowed comparison of the tissue accumulation and therapeutic selectivities of agents with different photophysical and photobiological properties. For this purpose three tests (fluorescence detection, therapeutic and histopathological) have been performed.

LIF measurements were performed to determine an optimal time between injection and photoirradiation and revealed different kinetics of tissue accumulation for the agents tested (Figure 2). In the model used the LIF signals collected from the foot with the tumour were composite signals from the skin and underlying tumour, whereas in the normal foot the signal was obtained from the skin and underlying muscles. Although the fluorescence signal from the tumour was attenuated by the skin, the kinetic patterns

**Figure 6** Tumour growth inhibition ratio (in per cent as compared with untreated control) as a function of light dose after PDT with Chl ■, PII □ and ALA ◆.

**Figure 7** Normal foot oedema expression after PDT with different light doses. (**a**) ALA (200 mg $kg^{-1}$) was injected 1 h before photoirradiation. (**b**) Chl (5mg $kg^{-1}$) was injected 3 h before photoirradiation. (**c**) PII (10 mg $kg^{-1}$) was injected 24 h before photoirradiation. Eight animals in each group were used. Error bars = s.e. ■, 300 J $cm^{-2}$; ●, 240 J $cm^{-2}$; △, 180 J $cm^{-2}$; ▲, 120 J $cm^{-2}$.

observed reflected real concentration changes in the tissues. The kinetic pattern of ALA-induced PpIX production was characterised by rapid accumulation in tumour and slower accumulation in normal tissues (Figure 2a). As a result, the maximal tumour–normal tissue ratio of porphyrin accumulation was registered 1 h after injection of ALA, followed by a rapid decrease in selectivity. In contrast, PII was retained by the tumour, and selectivity of tumour accumulation compared with normal tissue reached maximal values at 48–72 h (Figure 2c). After injection of Chl, rapid accumulation and removal in the tumour and in the normal tissues was observed; however, the fluorescence signal in the foot with the tumour was significantly higher than in the normal foot

**Figure 8** Normal tissue damage index as a function of light dose after PDT with Chl ■, PII ◆ and ALA □.

during the first 24 h (Figure 2b). The selectivity of porphyrin accumulation in the tumour at the time of photoirradiation obtained by the LIF measurements was in the order ALA > Chl > PII (Table I).

Results of the PDT study using photoirradiation by HIPLS revealed that both the anti-tumour effect and the normal tissue damage index were enhanced with increasing light doses for all agents (Figures 6 and 8). In order to correctly compare PDT selectivity, an assessment of the normal tissue damage index per equal anti-tumour effect (TGIR) was performed. This evaluation is based on the regression analysis of normal and tumour tissue responses to PDT as a function of the applied light dose. Such an assessment can be performed at the optimal regimen for each PDT agent and disregards the differences in administered doses (tissue concentrations) and differences in their photophysical properties.

The results of the present study showed that with the treatment regimens used Chl was more effective than PII and ALA-induced PpIX for inhibition of tumour growth (Figure 6), and caused minimal damage (expression of oedema value) to normal tissue (Table I). Therapeutic selectivity of the agents at minimal values of anti-tumour effect (TGIR 50%) was in the order Chl > PII > ALA, but the order Chl > ALA > PII was obtained when the anti-tumour effect was more significant (TGIR 80% and TGIR 100%). This was the result of more prolonged normal foot oedema after PII-PDT than after Chl-PDT or ALA-PDT, when high doses of photoirradiation were applied (Figure 7). These observations can reflect the peculiarities in biological properties of the agents (pharmacokinetics and tissue distribution, character of tissue damage effects, etc).

The difference in PDT selectivity observed between Chl and PII correlates well with the LIF test, which showed a higher tumour–normal tissue accumulation ratio at the moment of treatment for Chl than for PII. It has been shown that the mechanism of anti-tumour effect after PDT with chlorins (Chl or NPe6) and PII is similar and based on damage to the microvasculature and blood circulation disturbances leading to tumour necrosis (Kostenich *et al.*, 1991; Nelson *et al.*, 1988). Nevertheless, different ultrastructural changes in the subendothelial zone, and fragmentation (NPe6) *vs* coalescence (PII) of collagen fibres has been found (Nelson *et al.*, 1988). It has also been shown that damage to the endothelial cells (with a subsequent release of the vasoactive compounds provoking vessel constriction) and macromolecular leakage from venules were observed after PDT with PII (Fingar *et al.*, 1990, 1992). In contrast, no vessel constriction and minimal macromolecular leakage from venules was noted after PDT with NPe6 (McMahon *et al.*, 1994). We suggest that the same differences in PDT effects can occur between Chl and PII. Although blood circulation disturbances and haemostasis, resulting in tissue anoxia and nutritional deficiency, are probably the dominant factors responsible for tumour necrosis after PDT with chlorins and

PII, other mechanisms such as direct cytotoxic effects have also been shown for Chl and NPe6 and may be involved in tumour response to PDT (Kostenich et al., 1991; McMahon et al., 1994). Histological examination carried out in the present study revealed that blood stasis, oedema and haemorrhages occurred in normal and tumour tissues after PDT with both agents. However, certain differences have been found: PII-PDT, as compared with Chl-PDT, caused more pronounced damage to epidermis, induced more severe oedema and subepidermal blister formation, caused damage to the blood vessel walls and induced thrombi formation (Figures 3 and 4).

ALA-PDT has a different systemic mechanism than either Chl or PII. The endogenous route of ALA-induced PpIX production presupposes that mitochondrial damage leads to direct cell killing (Malik and Lugaci, 1987). The LIF test data showed the highest tumour–normal accumulation ratio for ALA-induced PpIX in our tumour model. Enhanced PDT selectivity was expected after ALA administration, but in fact was not found at low light doses. The results demonstrated that oedema of the normal foot after ALA-PDT with light doses of 120–180 J $cm^{-2}$ was more pronounced than after Chl-PDT or PII-PDT, but when the light doses increased, normal tissue damage was less than for PII-PDT (Figure 8, Table I). At the high light doses the selectivity was higher for ALA-PDT than for PII-PDT.

We suggest that these results can be explained by the histological examinations, which showed that the epidermis was extensively damaged after ALA-PDT, as compared with Chl and PII (Figures 3 and 4). This is consistent with previous findings revealing increased PpIX fluorescence in the epidermis and high skin photosensitivity after ALA administration (Peng et al., 1992; Divaris et al, 1990). During photoirradiation the relatively thin epidermis layer receives the highest light dose in addition to the preferential production of PpIX occurring in this layer. Therefore, even at low light doses epidermal necrosis and subsequent acute inflammatory reaction can result in pronounced oedema.

In the tumour, some of the morphological changes after ALA-PDT were quite different from those observed after PDT with Chl or PII and could be attributed to direct parenchymal tumour cell kill with slight damage to tumour vasculature. Moderate oedema with significant granulocyte infiltration observed after treatment is the indirect proof of preserved blood circulation in the tissue. Nevertheless, vascular damage during ALA-PDT cannot be excluded at certain light doses, because systemic administration of ALA can induce enhanced PpIX synthesis in the endothelial cells. This fact is based on the observation of blood stasis after photoirradiation with doses of 240 and 300 J $cm^{-2}$.

In conclusion, it is suggested that selectivity of PDT for different photosensitisers may be determined by biological mechanisms of action such as agent-specific interstitial distribution (binding sites) and the characteristics of tissue damage effects. The results of the present study indicate that integrated information obtained from a LIF study, a PDT selectivity test and a histopathology examination is useful in the evaluation of different agents. The experimental model and approach described can be used to assess new potential photosensitisers.

**Acknowledgements**
This study was supported by Energy Systems Co., which provided the Photodyne light system and skilled technical assistance.

### References

BEDWELL J, MACROBERT AJ, PHILLIPS D AND BOWN SG. (1992). Fluorescence distribution and photodynamic effect of ALA-induced PpIX in the DMH rat colonic tumour model. *Br. J. Cancer*, **65**, 818–824.

CAIRNDUFF F, STRINGER MR, HUDSON EJ, ASH DV AND BROWN SB. (1994). Superficial photodynamic therapy with topical 5-aminolaevulinic acid for superficial primary and secondary skin cancer. *Br. J. Cancer*, **69**, 605–608.

DIVARIS DXG, KENNEDY JC AND POTTIER RH. (1990). Phototoxic damage to sebaceous glands and hair follicles of mice following systemic administration of 5-aminolevulinic acid correlates with localized protoporphyrin IX fluorescence. *Am. J. Pathol.*, **136**, 891–897.

EVENSEN JF AND MOAN J. (1987). A test of different photosensitizers for photodynamic treatment of cancer in a murine tumour model. *Photochem. Photobiol.*, **46**, 859–865.

FINGAR VH, WIEMAN TJ, WIEHLE SA AND CERRITO PB. (1990). The role of tromboxane and prostacyclin release on photodynamic-therapy-induced tumour destruction. *Cancer Res.*, **50**, 2599–2603.

FINGAR VH, WIEMAN TJ, WIEHLE SA AND CERRITO PB. (1992). The role of microvascular damage in photodynamic therapy: the effect of treatment on vessel constriction, permeability and leukocyte adhesion. *Cancer Res.*, **52**, 4914–4921.

FRISOLI JK, TUDOR EG, FLOTTE TJ, HASAN T, DEUTSCH TF AND SCHOMACKER KT. (1993). Pharmacokinetics of a fluorescent drug using laser-induced fluorescence. *Cancer Res.*, **53**, 5954–5961.

GOMER CJ. (1991). Preclinical examination of first and second generation of photosensitisers used in photodynamic therapy. *Photochem. Photobiol.*, **54**, 1093–1107.

GOMER CJ AND FERRARIO A. (1990). Tissue distribution and photosensitising properties of mono-L-aspartyl chlorin e6 in a mouse tumour model. *Cancer Res.*, **50**, 3985–3990.

KENNEDY JC AND POTTIER RH. (1992). Endogenous protoporphyrin IX, a clinically useful photosensitiser for photodynamic therapy. *J. Photochem. Photobiol., B,Biol.*, **14**, 275–292.

KENNEDY JC, POTTIER RH AND PROSS DC. (1990). Photodynamic therapy with endogenous protoporphyrin IX: basic principles and present clinical experience. *J. Photochem. Photobiol., B,Biol.*, **6**, 143–148.

KENNEDY JC, NADEAU P, PETRYKA ZJ, POTTIER RH AND WEAGLE G. (1992). Clearance times of porphyrin derivatives from mice as measured by in vivo fluorescence spectroscopy. *Photochem. Photobiol.*, **55**, 729–734.

KOSTENICH GA, ZHURAVKIN IN, FURMANCHUK AV AND ZHAVRID EA. (1991). Photodynamic therapy with chlorin $e_6$. A morphologic study of tumour damage efficiency in experiment. *J. Photochem. Photobiol., B,Biol.*, **11**, 307–318.

KOSTENICH GA, ZHURAVKIN IN, FURMANCHUK AV AND ZHAVRID EA. (1993). Sensitivity of different rat tumour strains to photodynamic treatment with chlorin $e_6$. *J. Photochem. Photobiol., B,*Biol., **17**, 187–194.

KOSTENICH GA, ZHURAVKIN IN AND ZHAVRID EA. (1994). Experimental grounds for using chlorin $e_6$ in the photodynamic therapy of malignant tumours. *J. Photochem. Photobiol., B,Biol.*, **22**, 211–217.

MCMAHON KS, WIEMAN TJ, MOORE PH AND FINGAR VH. (1994). Effects of photodynamic therapy using mono-L-aspartyl chlorin e6 on vessel constriction, vessel leakage, and tumour response. *Cancer Res.*, **54**, 5374–5379.

MALIK Z AND LUGACI H. (1987). Accumulation and translocation of endogenous-porphyrins: their relation to photodynamic destruction of human leukemic cells by photoactivation of endogenous porphyrins. *Br. J. Cancer*, **56**, 589–595.

MALIK Z, KOSTENICH G, ROITMAN L, EHRENBERG B AND ORENSTEIN A. (1995). Topical application of 5-aminolaevulinic acid, DMSO and EDTA: protoporphyrin IX accumulation in skin and tumours of mice. *J. Photochem. Photobiol., B. Biol.*, **28**, 213–218.

NELSON JS, LIAW LH, ORENSTEIN A, ROBERTS WG AND BERNS MW. (1988). Mechanism of tumour destruction following photodynamic therapy with hematoporphyrin derivative, chlorin, and phthalocyanine. *J. Natl Cancer Inst.*, **80**, 1599–1605.

ORENSTEIN A, KOSTENICH G, ROITMAN L, TSUR H, KATANICK D, KOPOLOVIC J, EHRENBERG B AND MALIK Z. (1996). Photodynamic therapy of malignant lesions of the skin mediated by topical application of 5-aminolaevulinic acid in combination with DMSO and EDTA. *Lasers Life Sci.*, **7**, 1–9.

PENG Q, MOAN J, WARLOE T, NESLAND M AND RIMINGTON C. (1992). Distribution and photosensitising efficiency of porphyrins induced by application of exogenous 5-aminolaevulinic acid in mice bearing mammary carcinoma. *Int. J. Cancer*, **52**, 433-443.

REGULA J, RAVI B, BEDWELL J, MACROBERT AJ AND BOWN SG. (1994). Photodynamic therapy using 5-aminolaevulinic acid for experimental pancreatic cancer-prolonged animal survival. *Br. J. Cancer*, **70**, 248-254.

SPIKES JD. (1990). Chlorins as photosensitisers in biology and medicine. *J. Photochem. Photobiol.*, B,Biol., **6**, 259-274.

SPIKES JD. (1993). Photosensitising properties of mono-L-aspartyl chlorin $e_6$ (NPe6): a candidate sensitiser for the photodynamic therapy of tumours. *J. Photochem. Photobiol.*, B,Biol., **17**, 135-143.

SVANBERG K, ANDERSSON T, KILLANDER D, WANG I, STENRAM U, ANDERSSON-ENGELS S, BERG R, JOHANSSON J AND SVANBERG S. (1994). Photodynamic therapy of non-melanoma malignant tumours of the skin using topical delta-aminolaevulinic acid sensitisation and laser irradiation. *Br. J. Dermatol.*, **130**, 743-751.

ZHURAVKIN IN, KOSTENICH GA AND ZHAVRID EA. (1992). Photodynamic activity of chlorin $e_6$ in experiment. In *Photodynamic Therapy and Biomedical Lasers*, Spinelli P, Dal Fante M and Marchesini R. (eds) p. 535. Elsevier Science Publishers: Amsterdam.